# Magnetic Resonance Imaging Evaluation of Changes in Rectus Abdominis Muscles after High-Intensity Focused Electromagnetic Therapy-a pilot study for Asians

**DOI:** 10.7150/ijms.102954

**Published:** 2024-10-28

**Authors:** Hao-Yun Liu, Caleb Yu, Yao-Ting Lee, Chia-Hung Chang, Tsen-Fang Tsai, Shyh-Jye Chen

**Affiliations:** 1 Department of Medical Imaging, National Taiwan University Hospital Hsin-Chu Branch, Hsin-Chu, Taiwan.; 2 Department of Medical Imaging, National Taiwan University Hospital and Children Hospital, National Taiwan University, Taipei, Taiwan.; 3 Department of Dermatology, National Taiwan University Hospital and National Taiwan University College of Medicine, Taipei, Taiwan.

## Abstract

**Purpose:** This study aimed to evaluate the effects of high-intensity focused electromagnetic (HIFEM) technology on rectus abdominis muscles (RAMs) in Asian individuals, hypothesizing that HIFEM is safe and effective for body contouring in this population and that volumetric assessment provides more accurate results than thickness measurements alone.

**Methods:** In this prospective, single-center pilot study, six Asian participants (3 males, 3 females; mean age 45.5 years) underwent HIFEM training. Magnetic resonance imaging (MRI) was performed before treatment and at 2- and 3-months post-treatment to assess RAM volume and thickness. The DIXON VIBE sequence was used for high-resolution imaging, and 3D segmentation tools were employed for precise measurements.

**Results:** All participants showed increased RAM volume after 3-months treatment, with percentage increases ranging from 4.0% to 21.1%. Thickness measurements were inconsistent, with some participants experiencing decreases. No adverse events were reported during the study period.

**Conclusion:** This pioneering study establishes the safety and effectiveness of HIFEM technology for RAM training in Asian individuals. The discrepancy between volume and thickness changes highlights the importance of volumetric assessment in evaluating muscle remodeling. While limited by small sample size and short follow-up, this research provides a foundation for further investigation of HIFEM technology in diverse populations and underscores its potential as a non-invasive approach in aesthetic medicine.

## Introduction

The field of body contouring has witnessed substantial growth over recent decades. Liposuction emerged in the 1980s as a pivotal surgical method for fat removal, marking a significant advancement in aesthetic surgery^1^. In recent years, the advent of non-invasive techniques such as cryolipolysis, radiofrequency, low-level laser therapy, and focused ultrasound has transformed the landscape of fat reduction, offering safer and effective alternatives to traditional surgery^2^. Despite their efficacy in targeting adipose tissue, these methods often overlook the importance of muscular composition in defining aesthetic appearance. Traditional muscle enhancement approaches have been largely limited to physical exercise regimens. The integration of innovative techniques that concurrently address fat reduction and muscle enhancement holds promise for more comprehensive solutions in achieving desired body contours. This dual-focused approach could revolutionize body contouring, providing patients with more holistic and satisfactory outcomes.

Since 2000, nonvolitional electrical and electromagnetic muscle stimulation has been employed therapeutically to aid injury recovery, strengthen skeletal muscle, and prevent muscle atrophy^3^-^6^. The non-thermal and non-invasive nature of this technology ensured its safety for human use. Despite its efficacy, the technology was not widely adopted at the time. This changed in 2018 with the introduction of high-intensity focused electromagnetic (HIFEM) technology for non-invasive muscle building^7^. HIFEM technology represents a significant advancement, providing a novel, non-thermal approach to stimulate muscle growth and reduce fat layers simultaneously. Although the principle of electromagnetic induction underpinning HIFEM is not entirely new, its application in cosmetic medicine for body contouring is innovative. This dual-benefit approach of muscle toning and fat reduction offers a promising new avenue in aesthetic medicine. The integration of HIFEM technology into clinical practice marks a substantial shift, addressing both muscle enhancement and fat reduction, thereby providing a more holistic solution for patients seeking improved body contours. As the technology evolves, its potential to revolutionize body contouring and muscle conditioning continues to expand, heralding a new era in non-invasive aesthetic treatments.

Several studies have investigated the effects of high-intensity focused electromagnetic (HIFEM) technology on the remodeling of rectus abdominis muscles (RAMs)^8^-^14^. These studies primarily evaluated outcomes based on muscle length. However, relying solely on length can introduce bias due to allometry, where tissue growth does not occur in equal proportions^15^. This study aims to provide a quantitative analysis of RAM shape remodeling by assessing tissue volume following HIFEM training. We hypothesize that HIFEM training is safe and effective for body shaping and contouring in Asian populations, and that tissue volume evaluation offers a more accurate assessment than thickness alone.

## Methods

### Study population

This prospective, single-center, non-randomized pilot study included six research subjects, comprising three females and three males, with an average age of 45.5 years and a mean Body Mass Index (BMI) of 21.55 kg/m^2 prior to undergoing high-intensity focused electromagnetic (HIFEM) training. Exclusion criteria encompassed (1) pregnancy or planning to become pregnant; (2) any medical condition contraindicating the use of electromagnetic fields; (3) cardiac issues or implanted cardiac devices; (4) unhealed abdominal wounds or recent abdominal surgery; (5) use of medications known to influence weight or muscle mass. Detailed baseline demographic profiles of the participants were meticulously recorded. Informed consent was obtained from all subjects, who were not financially compensated for their participation or completion of the study. The research adhered to the relevant ethical standards and was conducted under a protocol approved by the hospital's Research Ethics Committee (202207218RIFA).

### Study design

According to the Research Ethics Committee-approved protocol, all research participants were instructed to continue with their usual diet and activity levels, making no changes until this study was concluded. The HIFEM technology device (CM Slim, MM Medical Aesthetics, Australian) was used for the participants under the protocol. During the study period, all participants underwent HIFEM training program and MRI was performed before and 2, 3 months after HIFEM training. Any adverse events after training are also recorded.

### HIFEM training program (CM Slim)

The device automatically varied the output intensity, frequency, output wave pattern, and duration for the stimulation, to maximize the effect of the treatment as accordance to the gender and the anatomy area being treated. The user could further select the following modes as accordance to the desired effect: 1) High Intensity for a simulated Hi-Intensity Interval Training (HIIT), 2) Muscle Toning for a series of rapid stimulation that is designed to firm up muscle for clearer muscle definition, 3) Strength mode for a series of longer duration, higher intensity stimulation that is designed to increase muscle mass and strength, 4) Combo 1 for a combination of High Intensity and Muscle Toning, and 5) Combo2 for a combination of Muscle Toning and Strength.

**Figure 1:**
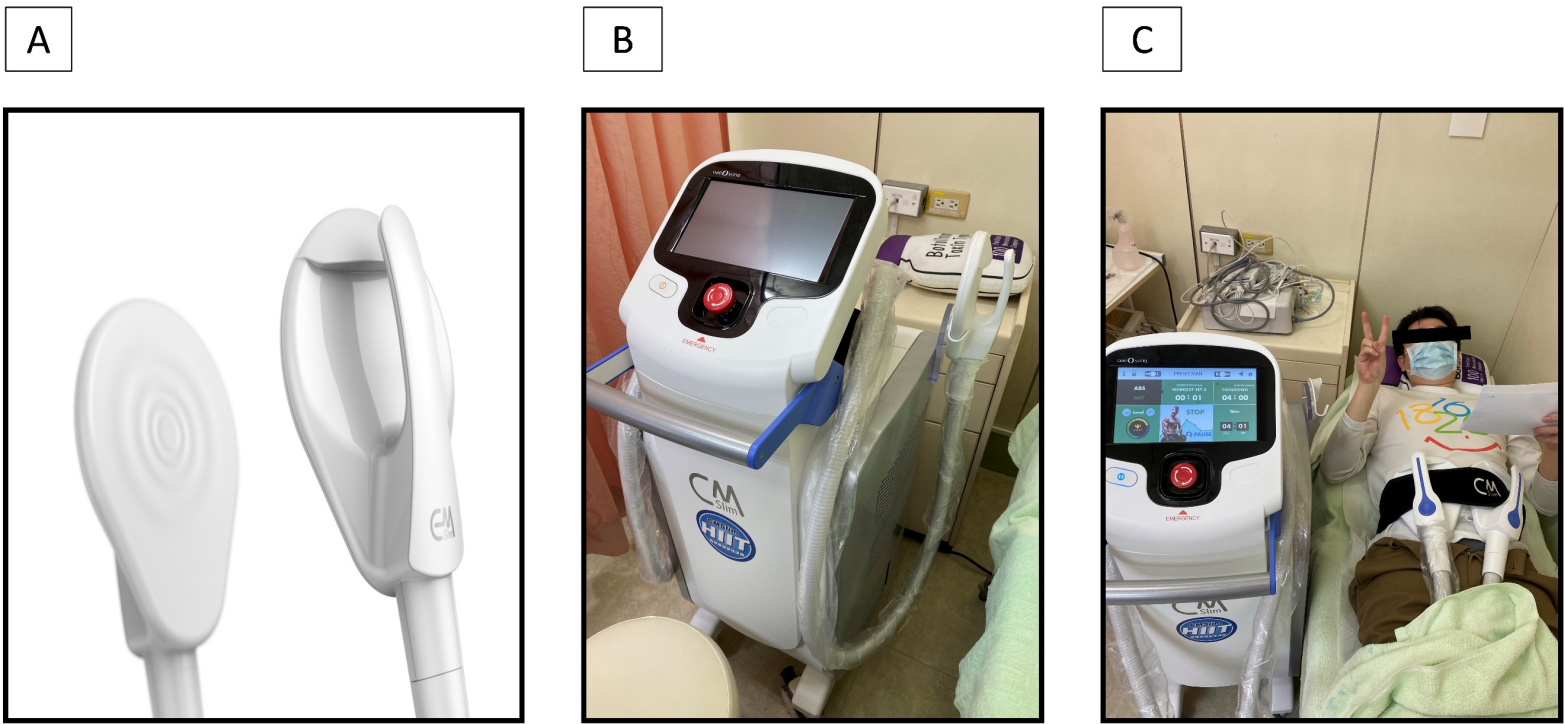
HIFEM training unit. (A) The applicator of CM slim; (B) The appearance of CM slim; (C) actual training situation of CM slim to the participant.

Participants did not receive any anesthesia and were placed in the supine position. Two applicators (Figure 1) were placed on the abdomen at the umbilical level. Training regimen is selected as accordance to the gender of the participant, then “Abs” as the focus anatomy, and Combo1 as the training mode. The operator controlled the stimulation intensity, started level 1 and slowly stepped up the until participants' subjective tolerance threshold as vocally cued by the participants. The procedure than would continue to its termination at this intensity level, or if the participant expressed excessive discomfort.

### MRI examination

**Figure 2:**
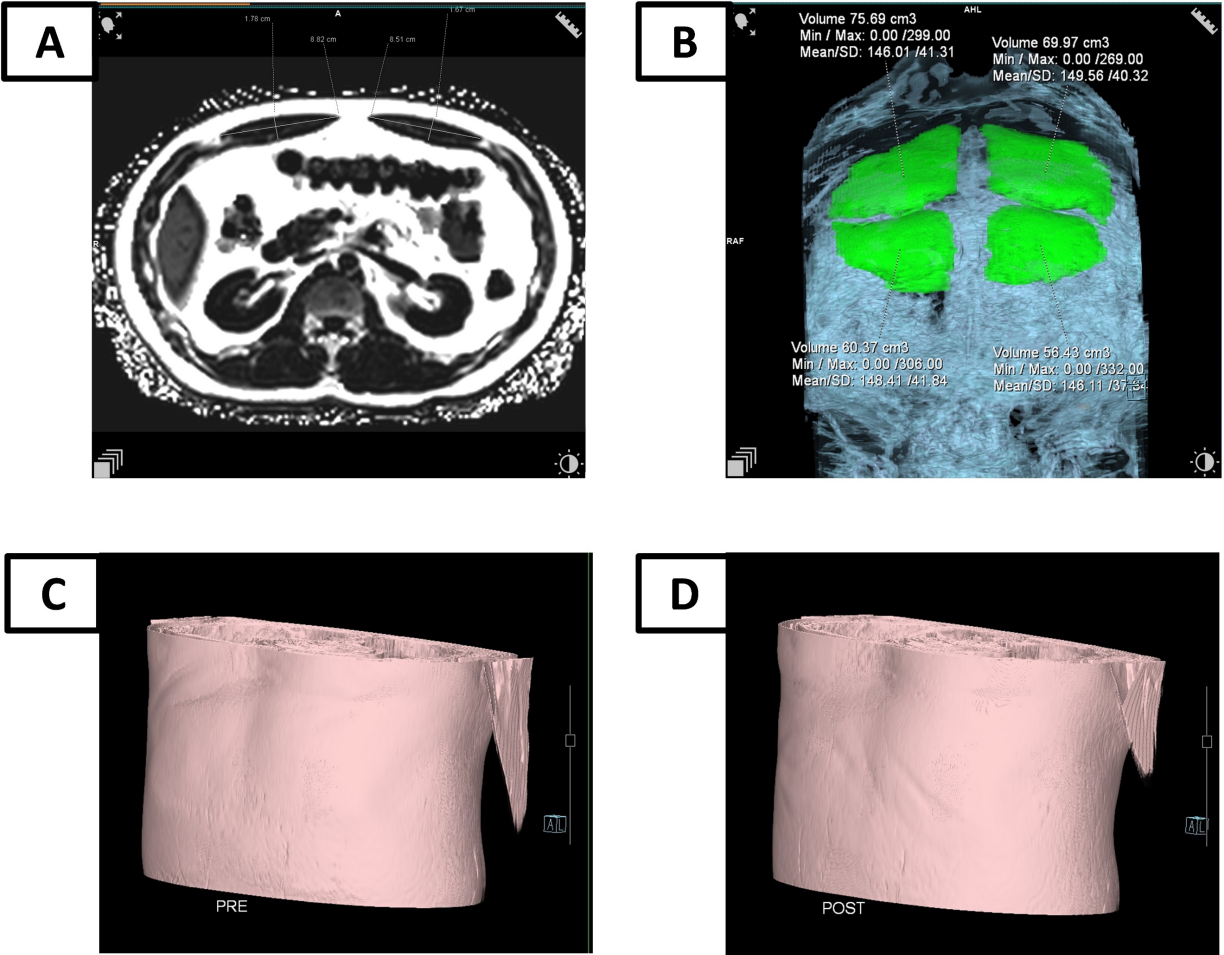
The MRI examination for RAM. (A) The thickness evaluation by MRI; (B) The volume evaluation by MRI; (C) MRI simulation of abdominal morphology before HIFEM training; (D) MRI simulation of abdominal morphology three months after HIFEM training.

The MRI examination employed the DIXON VIBE sequence to obtain images in the sagittal orientation, covering the abdomen and pelvis with a field of view (FOV) of 500 mm and a voxel size of 0.8x0.8x3.0 mm. Post-imaging processing was conducted using the Siemens syngo MR XA31 workstation. The analysis utilized MR View&GO's 3D imaging tools, specifically the Region Growing Segmentation Tools within the 3D Editing module, to achieve precise measurements. This protocol ensured high-resolution imaging, essential for detailed anatomical assessment, by leveraging the DIXON VIBE sequence's capabilities for fat suppression and tissue contrast enhancement. The Siemens syngo MR XA31 software facilitated efficient post-processing, enabling comprehensive analysis through its advanced 3D tools. The high-resolution images provided a clear view of the anatomical structures, allowing for accurate volume measurements of the rectus abdominis muscles. This imaging protocol was instrumental in the study, ensuring that the data collected was both precise and reliable. The use of the Region Growing Segmentation Tools within the 3D Editing module allowed for meticulous evaluation of muscle tissue changes pre- and post-HIFEM training, as shown in figure 2.

The percentage of fat within the abdominal muscles was measured using axial Q-Dixon fat fraction (FF) imaging. Regions of interest (ROI) were delineated around each individual abdominal muscle to quantify the fat content. Additionally, during the follow-up period, visceral fat was measured. The measurement method involved determining the cross-sectional area of visceral fat on MRI by measuring downward from the midpoint of the rectus abdominis muscle.

### Statistical analysis

Statistical analyses were performed using Statistical Analysis System version 9.4 software (SAS Institute Inc., Cary, NC, USA). Descriptive statistics were calculated for all measured parameters. Changes in RAM measurements from baseline to 2- and 3-months post-treatment were analyzed using paired t-tests. A p-value < 0.05 was considered statistically significant.

The percentage difference for each parameter was calculated using the formula: Percentage difference = [(post-treatment value - Baseline value) / Baseline value] × 100%.

## Results

### Participant characteristics

Six Asian participants were enrolled in the study, comprising three males and three females, with ages ranging from 32 to 63 years. Prior to HIFEM training, all participants underwent MRI assessment to evaluate the condition of their RAM. The RAM volume ranged from 75.0 to 234.2 cm³, while the RAM thickness ranged from 8.5 to 16.4 mm. Detailed baseline characteristics and measurements are presented in Table 1.

### Muscle change measurement by MRI following

The evaluation of RAM changes in all participants is documented in Table 2. Follow-up assessments were conducted at 2 and 3 months post-HIFEM training, evaluating thickness, volume, and percentage difference. Volume evaluations revealed an increase in RAM volume for all six participants at both the 2nd and 3rd months, with percentage increases ranging from 0.6% to 21.1%. Conversely, thickness evaluations indicated that some participants experienced a decrease in RAM thickness, with changes ranging from -12.2% to -0.9%. These findings suggest that while volume measurements showed a consistent increase, thickness measurements did not. Notably, no adverse events were reported among the six participants during the training period. One participant (No. 3) developed moderate muscle soreness after training, which resolved on its own within two days with no further discomfort. The other five participants experienced mild soreness, which subsided shortly after training. Overall, the muscle soreness caused by HIFEM training was short-lived and within an acceptable range for all six participants.

### Fat change measurement by MRI following

Changes in RAM fat and visceral fat areas among all participants are documented in Table 2. After three months of HIFEM training, over half of the participants showed a mild decrease in RAM fat percentage. Conversely, visceral fat exhibited an increasing trend. Moreover, the evaluation indicated that the rate of increase in visceral fat was significantly higher than the decrease in RAM fat percentage (0.06% increase in RAM fat vs. 2.73% increase in visceral fat). These results support the hypothesis that HIFEM training may reduce RAM fat, while visceral fat remains unaffected.

## Discussion

To our knowledge, this is the first study of HIFEM technology used on Asians. This study demonstrated the safety and effective of HIFEM technology for RAM training. Not one participant felt any discomfort during the training period, nor did any complain about muscle soreness after the training program. The result showed that volume evaluation is better than the thickness for muscle training by HIFEM. Moreover, this study also showed the suitable of HIFEM technology for Asians.

HIFEM technology is not new, but its practical applications have only emerged in the last two decades. Although several studies have explored the use of HIFEM technology in body contouring, only a few have utilized imaging techniques for precise measurement^10^,^12^-^14^. These studies provide valuable insights into the effectiveness of HIFEM technology in enhancing muscle tone and reducing fat layers. By employing advanced imaging modalities, such as MRI and ultrasound, these studies have demonstrated quantifiable changes in muscle volume and thickness, underscoring the potential of HIFEM technology in non-invasive body contouring. Table 4 is the list for the above studies, which highlights that our research uniquely focuses on an entirely Asian cohort, while other studies primarily involve Western participants. Three months post-HIFEM training, the imaging evaluations of abdominal muscle measurements reveal that Western participants exhibit a significant increase in muscle thickness, whereas such differences are not observed in Asian participants. This discrepancy might indicate three key points: first, there may be racial differences in the proportion of muscle thickness increase; second, thickness alone may not effectively capture the benefits of HIFEM training; third, the biological factors, such as muscle adaptation, fluid retention, BMI…etc., might cause inconsistencies between muscle thickness and volume. As Nevill *et al.* have noted, muscle growth is not always proportional, and changes during training might affect width rather than thickness^15^. Therefore, when assessing muscle changes, volume measurements may offer a more accurate representation than thickness alone, potentially reducing measurement errors. This approach is essential for accurately evaluating the efficacy of HIFEM technology in muscle toning and body contouring across diverse populations.

In some studies, body changes have been measured using photographic methods^8^,^11^. From a cosmetic medicine perspective, this approach may suffice. However, from a scientific standpoint on muscle training, non-invasive imaging techniques are necessary. Ultrasound, computed tomography, or MRI are more appropriate tools for this purpose. Specifically, for precise measurement, assessing muscle volume is more accurate than measuring muscle thickness. Non-invasive imaging not only provides a detailed and quantifiable evaluation of muscle morphology but also ensures a more reliable and reproducible assessment of training outcomes. Hence, adopting these advanced imaging modalities is crucial for accurately monitoring and validating the effects of muscle training interventions. In our opinion, MRI is the optimal tool for measuring training outcomes because it can evaluate both muscle volume and muscle fat content. The earlier review showed the value of muscle measure by MRI^16^. Recently, this method is still be used by researchers^17^,^18^. This method provides greater accuracy compared to other measurement tools. In our pilot study, aside from the small sample size, we did not control for biological variables when initially recruiting participants. As a result, it was difficult to eliminate potential confounding factors. Future research should place greater emphasis on participant selection from the start to minimize differences and reduce variability among participants.

One clinical issue associated with aging is sarcopenia, a condition characterized by the loss of skeletal muscle mass and strength, which was first proposed by Irwin Rosenberg^19^. Sarcopenia primarily affects the elderly, increasing their risk of injury due to a higher incidence of falls^20^. Some systemic diseases, such as inflammatory bowel disease, also cause sarcopenia^21^. Resistance exercise is one effective method to slow the symptoms of sarcopenia^22^. however, the dropout rate from these training programs exceeds 30%, as older age and poorer physical performance are correlated with higher dropout rates^23^. Recent studies have shown positive outcomes in combating sarcopenia using electrical stimulation technology^24^-^26^​​. Unlike HIFEM technology, which uses electromagnetic fields to generate electrical currents within muscle tissue, electrical stimulation delivers direct electrical currents via skin electrodes. Electrical stimulation is limited in its ability to affect deeper muscle tissues, whereas HIFEM can target these areas effectively. HIFEM technology may offer a safer, more effective, and more acceptable alternative for alleviating sarcopenia symptoms^27^. However, further studies are needed to validate this approach​​.

While this study provides valuable insights into the effects of HIFEM therapy on rectus abdominis muscles in an Asian population, several limitations should be acknowledged. First, Study Design Limitations: the small sample size (n=6) limits the statistical power and generalizability of our findings. The lack of a control group makes it challenging to rule out the influence of confounding factors. As a single-center study, there may be institutional bias. The non-randomized selection of participants could introduce selection bias. Additionally, the absence of blinding for both researchers and participants may lead to potential bias in the results. Second, time Frame Limitation: the short-term follow-up period of only three months post-treatment precludes assessment of long-term effects and the sustainability of observed changes. Furthermore, it remains unclear whether the volumetric muscle changes are sustained over time or if fat reduction continues. Third, assessment Methodology Limitations: our evaluation primarily focused on muscle volume and thickness, potentially overlooking other important parameters. The lack of functional assessment, such as muscle strength or performance measures, limits our understanding of the therapy's practical impact. Furthermore, without a functional assessment, such studies would be merely superficial rather than providing truly valuable clinical insights. Future studies should consider evaluating relevant parameters to strengthen their findings. Fourth, variable Control Limitation: the study did not strictly control for participants' diet and exercise habits, which could potentially influence the results and introduce confounding variables. At least, this study ensured that their meals were typical and not specifically tailored for muscle training. The last, economic Analysis Limitation: a cost-effectiveness analysis was not conducted, limiting our ability to evaluate the economic viability of this treatment method in clinical practice. These limitations highlight the need for future studies with larger sample sizes, randomized controlled designs, longer follow-up periods, and more comprehensive assessment methods to further validate and expand upon our findings. Despite these limitations, our study provides a valuable foundation for understanding the effects of HIFEM therapy in an Asian population and sets the stage for more extensive research in this area.

In conclusion, this pioneering study not only establishes the safety and effectiveness of HIFEM technology for RAM training in Asian individuals but also underscores the importance of appropriate assessment methods in evaluating treatment outcomes. The discrepancy between volume and thickness changes highlights the importance of volumetric assessment in evaluating muscle remodeling. These findings provide a foundation for further research into the application of HIFEM technology for body contouring in diverse populations and highlight the potential of this non-invasive approach in aesthetic medicine. Besides, the future researchers might pay more attention to the HIFEM application to sarcopenia.

## Figures and Tables

**Table 1:** The characteristic of participants

	Ethnicity	Gender	Age	Height (Cm)	Weight (Kg)		RAM baseline (before training)	
							RAM volume (cm^3^)	RAM thickness (mm)
No. 1	Asian	M	58	161	57		79.2	12.2
No. 2	Asian	M	45	173	77		234.2	16.4
No. 3	Asian	M	33	174	63		124.2	11.2
No. 4	Asian	F	63	160	62.5		98.3	8.5
No. 5	Asian	F	46	158	48		105.4	11.6
No. 6	Asian	F	32	171	56		75.0	13.3

**Table 2:** Changes in RAM during training period

**Thickness**	RAM baseline (before training)		2 Months after training				3 Months after training		
	RAM thickness (mm)		RAM thickness (mm)	Difference (%)	adverse events		RAM thickness (mm)	Difference (%)	adverse events
No. 1	12.2		13.0	+6.6%	No		12.6	+3.3%	No
No. 2	16.4		16.6	+1.2%	No		17.2	+4.9%	No
No. 3	11.2		11.4	+1.8%	No		11.9	+6.3%	No
No. 4	8.5		8.7	+2.4%	No		8.4	**-1.2%**	No
No. 5	11.6		11.5	**-0.9%**	No		10.3	**-11.2%**	No
No. 6	13.3		13.4	+0.8%	No		13.0	**-2.3%**	No
**Volume**	RAM baseline (before training)		2 Months after training				3 Months after training		
	RAM volume (cm^3^)		RAM volume (cm^3^)	Difference (%)	adverse events		RAM volume (cm^3^)	Difference (%)	adverse events
No. 1	79.2		95.3	+20.3%	No		95.9	+21.1%	No
No. 2	234.2		235.5	+0.6%	No		262.5	+12.1%	No
No. 3	124.2		134.8	+8.5%	No		129.1	+4.0%	No
No. 4	98.3		102.3	+4.2%	No		117.4	+19.4%	No
No. 5	105.4		117.3	+11.3%	No		121.4	+15.2%	No
No. 6	75.0		80.0	+6.7%	No		80.6	+7.5%	No

**Table 3:** Changes in RAM fat during training period

**RAM fat ratio**	Baseline (before training)		2 Months after training			3 Months after training	
	RAM fat ratio (%)		RAM fat ratio (%)	Difference (%)		RAM fat ratio (%)	Difference (%)
No.1	No data		4.3	-		3.2	-
No.2	2.4		2.4	0		2.2	-0.2%
No.3	3.2		3.3	+0.1%		3.7	+0.5%
No.4	0.9		2.0	+1.1%		1.9	+1.0%
No.5	3.4		4.8	+1.4%		3.3	-0.1%
No.6	2.7		3.0	+0.3%		1.8	-0.9%
**Average**				+0.58%			+0.06%
**Visceral fat area**	Baseline (before training)		2 Months after training			3 Months after training	
	Area (cm^2^)		Area (cm^2^)	Difference (%)		Area (cm^2^)	Difference (%)
No.1	143.5		168.4	+17.4%		164.9	+15.0%
No.2	233.1		238.0	+2.1%		226.4	-2.9%
No.3	87.8		91.4	+4.1%		91.7	+4.4%
No.4	48.4		47.4	-2.0%		43.7	-1.0%
No.5	251.0		267.5	+6.6%		258.9	+3.1%
No.6	55.4		50.7	-8.5%		54.2	-2.2%
**Average**				+3.28%			+2.73%

**Table 4:** Measurement data from published studies and present study of HIFEM training of the abdomen

Study (year)	No. patients	Ethnicity	Training device	Measure device	Baseline muscle thickness (mm, mean)	3 months training muscle thickness (mm, mean)
Jacob *et al.* (2020)^10^	10	Caucasian	Emsculpt	MRI	9.3	11.3
Jacob *et al.* (2021)^12^	40	Caucasian	Emsculpt	MRI	9.2	11.6
Samuels *et al.* (2022)^13^	40	Caucasian	Emsculpt-Neo	Ultrasound	9.4	11.7
Katz *et al.* (2023)^14^	3	Caucasian	Emsculpt-Neo	MRI	12.9	16.0
Current study	6	Asian	CM Slim	MRI	12.2	12.2

## Abbreviations

HIFEM: high-intensity focused electromagnetic
RAM: rectus abdominis muscle
MRI: magnetic resonance imaging
BMI: Body Mass Index
HIIT: Hi-Intensity Interval Training
FOV: field of view
FF: fat fraction
ROI: Regions of interest
